# Medical Society of the State of New York

**Published:** 1872-02

**Authors:** 


### Editorial.

### Medical Society of the State of New York.

The Society met on Tuesday, February 6th, at Albany. The meeting being called to order by the President, Dr. William C. Wey, of Elmira. Prayer was offered by Rev. Dr. Snively, which was followed by the President's Inaugural Address. He spoke of medical fellowship and the importance of medical ethics, suggesting it as a branch of medical education in schools. He referred to the tardy publication of the transactions, and of the rule adopted last year of allowing papers to be published in the Medical Journals. He suggested action to obtain room for the Annual Meetings of the Society in the new Capitol. He also referred to the spirit shown by physicians to prevent action by Medical Societies by appeal to the courts, and said that a course so subversive of morals and justice should be promptly met by prompt expulsion of the offender, and a cessation of social and professional relations with him. He urged the necessity of a law to protect from loss, physicians wrongfully prosecuted for malpractice on account of the irresponsible character of the plaintiff.

After paying a compliment to the medical literature of the State the address was closed by alluding to the only sad feature of the year the death of four prominent members, viz: Barnet B. Staats, of Albany, Andrew Van Dyck, of Oswego, Erastus L. Hart, of Elmira, and Henry D. Buckley, of New York. The inaugural was suggestive, well chosen and appropriate, and was listened to with much attention.

After the transaction of miscellaneous business Dr. Elliot, of New York, presented, in behalf of Dr. Sayer, a draft of a bill to be presented to the Legislature for the protection of physicians and surgeons in cases of alleged malpractice. The following papers were read; On Transitory Mania, by Dr. Cook, of Canandaigua, Objects of Medical Legislation, by Dr. Rogers, Chronic Cystitis, by Dr. Emmet, of New York. Chronic Inversion of the Uterus, by Dr. White, of Buffalo, Ophthalmic Cases, by Dr. Rider, of Rochester, and Surgery of Childhood, by Dr. Pooley, of Westchester. This closed the business of the day sessions, and not being able to attend the evening meeting we are unable to give any notice of the proceedings.

2nd day. The time of the morning was largely occupied by miscellaneons business. Dr. E. P. Gray, of Utica, read a very instructive paper on the Causation of Insanity, the following being a brief abstract:

He endeavored to prove the physical causation of insanity, first, by analogy. Infirmity or instability of elements is a predisposing cause of disease. This is especially true of nerve tissue, hence heredity is an important element of causation in insanity. This is a disease of the body and not of the mind.

He showed the insufficiency of moral causes to produce the mental disorder, except as they induce temper or prevent pathological changes, and that physical disease is a necessary factor. That age and sex are predisposing causes only when physiological processes are changed to pathological He spoke of the different theories of the origin of mind and its relation to the body, and in brief of mania transitoria or impulsive insanity.

In conclusion, and as showing the fact that physical causation is recognized by the most skilled and competent observers, he gave the classification of insanity as recommended by the Medico-Psychological Society of London and the International Congress of Alienists, which is based entirely upon this theory.

A lengthy report upon medical education was read by Dr. Squibb, and resolutions passed. The attractions of this day's proceedings were vested mainly in the President's Annual Address, and the banquet given at the Delevan House by the following gentlemen: Thomas Hun, M. D., J. V. P. Quackenbush, M. D., John Swinburne, M. D., Wm. H. Bailey, M. D., Chas. A. Robertson, M. D., W. C. Wey, M. D., Chas. H. Porter, M. D., J. R. Boulware, M. D., Levi Moore, M. D., Joseph Lewi, M. D., S. O. Vanderpoel, M. D., Wm. H. Craig, M. D., Edward R. Hun, M. D., Jacob S. Mosher, M. D., Amos Fowler, M. D.

A paper on Phonation by Dr. Moore, of Rochester, and a paper on Extra

Uterine Fœtation, by Dr. Jewett, of Canandaigua, we expect to publish in full in our Journal.

There were many valuable papers, and highly interesting discussions, which we are at present unable to even mention.

The meeting was of unusual interest, and every member appeared gratified and instructed. The Society are under many obligations to its officers who were eminently successful in conducting its affairs successfully and pleasantly.

The following is a partial report of the committee on nominations for the ensuing year, which was adopted:

*President*—Cornelius R. Agnew.

*Vice-President*—B. F. Sherman.

*Secretary*—Wm. H. Bailey.

*Treasurer*—Charles H. Porter.

*For Censors*—Southern District: Edward R. Squibb, Samuel T. Hubbard, H. C. Husted. Eastern District: James L. Babcock, P. McNaughton, C. C. Covell. Middle District: M. M. Bagg, Horace Lathrop, Charles G. Bacon Western District: J. F. Miner, C. C. Wyckoff, D. Colvin.

*Permanent Members*—First District: Nathan Bozeman, James L. Little. Second District: M. R. Holbrook, A. C. Hull. Third District: Leroy McLean, P. V. S. Pruyn. Fourth District: Arthur S. Wolf, Henry H. Green. Fifth District: G. L. Halsey, Edwin Hutchinson. Sixth District: Joshua B. Graves, J. H. Squire. Seventh District: C. G. Pomeroy, C. C. Murphy. Eighth District: Charles E. Rider.

*For Honorary Members*—M. Nelaton, Rudolph Virchow.

*Eligible for Honorary Membership*—Lewis Oakley, Elizabeth, N. J.; Charles H. Hewitt, Red Wing, Minn.; J. M Toner, Washington, D. C.; Joseph B. Brown, U. S. A.

*For the Degree of M. D.*—Cornelius H. Schaaps, Brooklyn; Wm. Lamont, Schoharie.

